# Changes in endotoxin levels in T2DM subjects on anti-diabetic therapies

**DOI:** 10.1186/1475-2840-8-20

**Published:** 2009-04-15

**Authors:** Omar S Al-Attas, Nasser M Al-Daghri, Khalid Al-Rubeaan, Nancy F da Silva, Shaun L Sabico, Sudhesh Kumar, Philip G McTernan, Alison L Harte

**Affiliations:** 1 Biochemistry Department, College of Science King Saud University Riyadh, KSA; 2 Diabetes Center, King Abdul-Aziz University Hospital, Riyadh, KSA; 3 University of Warwick, Warwick Medical School, Diabetes and Metabolism Unit, Coventry, CV4 7AL, UK

## Abstract

**Introduction:**

Chronic low-grade inflammation is a significant factor in the development of obesity associated diabetes. This is supported by recent studies suggesting endotoxin, derived from gut flora, may be key to the development of inflammation by stimulating the secretion of an adverse cytokine profile from adipose tissue.

**Aims:**

The study investigated the relationship between endotoxin and various metabolic parameters of diabetic patients to determine if anti-diabetic therapies exerted a significant effect on endotoxin levels and adipocytokine profiles.

**Methods:**

Fasting blood samples were collected from consenting Saudi Arabian patients (BMI: 30.2 ± (SD)5.6 kg/m^2^, n = 413), consisting of non-diabetics (ND: n = 67) and T2DM subjects (n = 346). The diabetics were divided into 5 subgroups based on their 1 year treatment regimes: diet-controlled (n = 36), metformin (n = 141), rosiglitazone (RSG: n = 22), a combined fixed dose of metformin/rosiglitazone (met/RSG n = 100) and insulin (n = 47). Lipid profiles, fasting plasma glucose, insulin, adiponectin, resistin, TNF-α, leptin, C-reactive protein (CRP) and endotoxin concentrations were determined.

**Results:**

Regression analyses revealed significant correlations between endotoxin levels and triglycerides (R^2 ^= 0.42; p < 0.0001); total cholesterol (R^2 ^= 0.10; p < 0.001), glucose (R^2 ^= 0.076; p < 0.001) and insulin (R^2 ^= 0.032; p < 0.001) in T2DM subjects. Endotoxin showed a strong inverse correlation with HDL-cholesterol (R^2 ^= 0.055; p < 0.001). Further, endotoxin levels were elevated in all of the treated diabetic subgroups compared with ND, with the RSG treated diabetics showing significantly lower endotoxin levels than all of the other treatment groups (ND: 4.2 ± 1.7 EU/ml, RSG: 5.6 ± 2.2 EU/ml). Both the met/RSG and RSG treated groups had significantly higher adiponectin levels than all the other groups, with the RSG group expressing the highest levels overall.

**Conclusion:**

We conclude that sub-clinical inflammation in T2DM may, in part, be mediated by circulating endotoxin. Furthermore, that whilst the endotoxin and adipocytokine profiles of diabetic patients treated with different therapies were comparable, the RSG group demonstrated significant differences in both adiponectin and endotoxin levels. We confirm an association between endotoxin and serum insulin and triglycerides and an inverse relationship with HDL. Lower endotoxin and higher adiponectin in the groups treated with RSG may be related and indicate another mechanism for the effect of RSG on insulin sensitivity.

## Introduction

In recent years, obesity, insulin resistance, as well as many of the features that comprise the metabolic syndrome, are associated with a low-grade, systemic, inflammatory condition [1,2]. In particular, the mediation of this sub-clinical inflammation in the pathogenesis of Type 2 Diabetes Mellitus (T2DM) is proposed to arise through increasing adiposity [3,4]; with adipose tissue representing a site of an acute phase response [5,6] through the production of known pro-inflammatory adipocytokines such as leptin, tumor necrosis factor (TNF-α) and interleukin (IL-6), amongst others [7-9]. Specifically, adipocytokines appear to have a duality of function, simultaneously mediating inflammation and insulin resistance through their effects on insulin action. Therefore, adipocytokines may function as a consequence of the cross-link proposed between metabolic and inflammatory pathways in adipocytes and immune cells [10-12].

Current therapies utilized in the treatment of T2DM include metformin and the thiazolidinediones (TZDs). These agents have principally been evaluated on the basis of their beneficial effects on glucose metabolism, due to their insulin enhancing properties. Metformin and rosiglitazone (RSG) are widely accepted first-line anti-diabetic drug therapies, whether taken as a monotherapy or in combination [13], and are considered effective with low incidence of hypoglycemia [14]. In addition to their effects on glucose homeostasis, metformin, itself, is known to reduce leptin, with resultant effects on inflammatory status and satiety [15]. Furthermore, the TZDs have previously been shown to have immunomodulatory effects [16]; reducing inflammation in both *in-vitro *and *in-vivo *models [17-19]. These findings highlight alternative pathways through which drug therapies are able to counteract the progressive nature of metabolic disease and their potential dual action on reducing obesity mediated T2DM.

Whilst metformin and the TZDs appear to offer dual functionality in insulin resistance and inflammation, the initial mediator for such an inflammatory insult is less well understood. One source of sub-clinical inflammation in patients with T2DM and coronary heart disease may arise through commensal bacteria derived from the gut, referred to as endotoxin [20]. Endotoxin is derived from lipopolysaccharide (LPS), which represents cell wall fragments of gram negative bacteria. Previous studies have demonstrated that subjects with obesity and T2DM have elevated circulating levels of endotoxin compared with non-diabetics (ND), which has been implicated in increased inflammatory risk [21]. Further support for this concept has arisen in subsequent studies where serum endotoxin levels are significantly higher in *ob/ob *and *db/db *mice compared with their normal weight counterparts [22]. Studies suggest elevated endotoxin levels may arise as a result of obesity related hyperinsulinemia; hence low-grade endotoxinemia may be caused by the effect of insulin on intestinal motility and/or intestinal permeability. In support of this theory, murine studies analyzing the gastrointestinal tract (GIT) of *ob/ob *and *db/db *mice identified pathological changes in the GIT cells. The findings indicated that insulin may act directly on the GIT to affect gut permeability and potentially increase endotoxin absorption [22]. Elevated circulating levels of endotoxin may then initiate an inflammatory response within adipose tissue, via innate immunity, in conjunction with the liver, as the latter is the primary site of endotoxin clearance under normal physiological circumstances [6,22]. This mechanism may, therefore, explain the state of sub-clinical inflammation often present in obese and type 2 diabetic subjects [2].

Whilst limited data have been presented on the increase of endotoxin in pathological conditions such as obesity and diabetes, no data, as yet, have evaluated the influence of insulin sensitizers, in combination, on inflammatory risk posed by circulating endotoxin. Whilst there is a large quantity of data available on obesity and T2DM in relation to the Western world, less information exists regarding populations in the transitional state. In particular, Saudi Arabia has experienced a rapid increase in wealth over a relatively short period of time as a consequence of the financial gains rendered by the oil industry, paralleled with swift industrialization and urbanization [23]. As a result, the burden of disease in Saudi Arabia is high. The Obesity Taskforce worldwide projections for 2030 predict that the incidence of diabetes will rise by 32% in Europe, 72% in the USA but will increase by a massive 164% in the Middle East [24,25]. As such, it is clear we need to have a fundamental understanding of their risk of inflammation and how insulin sensitizers, along with other therapies, may reduce such a risk.

Therefore, the aims of this present study were to establish any associations between endotoxin and insulin, glucose, lipid profiles and pro-inflammatory cytokines in a Saudi Arabian cohort consisting of obese, ND subjects and obese, T2DM patients on various anti-diabetic therapies. Furthermore, the study aimed to examine the influence of diet, metformin, RSG, met/RSG as well as insulin on endotoxin levels and its' association with inflammation. Ultimately, this study will evaluate whether alterations in endotoxin, coupled with altered metabolic profile, in type 2 diabetics might further explain the observed improvement in the clinical profiles of patients treated with these therapies. This present study, in combination with our previous findings, may therefore help determine endotoxin as a novel biomarker of T2DM and diabetic risk and hence proffer a new target for the treatment or prevention of diabetes.

## Methodology

This single-centre, prospective and cross-sectional study was carried out at the Diabetes Center of King Abdul-aziz University Hospital in Riyadh, Kingdom of Saudi Arabia. The study protocol was approved by the institutional review board and was conducted in accordance with the guidelines set by the College of Medicine and Research Center of King Saud University, Riyadh, Kingdom of Saudi Arabia ethics committee. All patients submitted written and informed consent prior to inclusion.

### Subjects

The study consisted of a total of 413 out-patients (male: 203; female: 210) age 20–80. Furthermore, 346 subjects were known type 2 diabetics, while the remaining 67 were ND, closely matched for BMI.

Inclusion criteria for the diabetics (prior diagnosis) were determined in the first screening visit and included HBA1c 6–11%, fasting plasma glucose 7.0–14.0 mmol/l; BMI 22–40 kg/m^2^; without co-existing diabetic complications, i.e. diabetic retinopathy, nephropathy, etc. Subjects must have received treatment (diet controlled, metformin, RSG, met/RSG or insulin) for at least one year. ND subjects had normal fasting plasma glucose (<5.6 mmol/l); HBA1c levels (4–6%) and were not taking any medications prior to commencement of research. Patients were excluded if they had poorly controlled diabetes with co-existing complications, smoking history, history of coronary heart disease and any unstable medical condition/s that would require immediate attention.

All subjects underwent a complete physical examination, which included an electrocardiogram prior to enrolment. Qualified patients were then stratified into 6 groups on the basis of their hypoglycemic therapies (diet-controlled (n = 36), metformin (n = 141), RSG (n = 22), met/RSG (n = 100) and insulin (n = 47)), in addition to the ND group (n = 67). Following the findings of the initial study, a sub-group of diabetic subjects (n = 11, BMI 31.7 ± 6.42 kg/m^2^) were treated with RSG for a period of 6 months (Post RSG: BMI 30.9 ± 4.42 kg/m^2^), in order to determine the specific effect of RSG treatment on endotoxin levels.

### In vivo Assessment of the Biochemical profile, Adipocytokines and Endotoxin Levels

On the assigned date, fasting blood samples were collected from participating subjects and lipid profiles and fasting plasma glucose determined using routine laboratory methods. Adipocytokine levels were also assessed using various sandwich enzyme-linked immunosorbent assays (ELISAs). These included adiponectin (Linco Ltd, USA; intra-assay variability 7.0%, interassay variability 8.4%), resistin (RnD Systems Ltd, UK; intra- 4.0%, interassay variability 7.7%), TNF-α (RnD Systems Ltd, UK; intra- 5.2%, interassay variability 7.4%), leptin (intra- 3.3%, interassay variability 5.4%) and C-reactive protein (CRP, Immunodiagnoztik AG, Germany; intra- 6%, interassay variability 11.6%). Insulin was analyzed via a solid phase enzyme amplified sensitivity immunoassay (Medgenix INS-ELISA, Biosource, Belgium). Lastly, endotoxin concentration was measured using a chromogenic kinetic limulus amebocyte assay (LAL assay, BioWhitaker, Walkersville MD), which had been validated, previously [21].

### Statistical Analyses

Data were analyzed using the Statistical Package SPSS for Windows, version 11.5. Data were expressed as mean ± standard deviation and mean (interquartile range) if not normally distributed. Groups were compared using ANOVA with Bonferroni adjustments for inter group comparisons. Simple and partial correlation coefficients between the variables were determined and multiple regression analysis was carried out to determine variables of interest. Triglycerides, insulin, leptin, adiponectin, CRP, resistin and endotoxin were logarithmically transformed to normalize data before correlations and statistical analyses were performed.

## Results

### Metabolic and Clinical Characteristics

#### Baseline characteristics

Table 1 shows the clinical and metabolic characteristics of the 6 groups analyzed in this study. The mean systolic blood pressures of the groups were similar, with the diet and RSG treated group having the lowest mean systolic blood pressures (111.6 ± 22.9 mmHg and 114.1 ± 28.7 mmHg, respectively) and the met/RSG group (128.4 ± 23.6 mmHg, p < 0.05) the highest. The mean diastolic blood pressures of the ND (84.1 ± 14.6 mmHg) and met/RSG treated groups (84.6 ± 13.0 mmHg) were significantly lower than the insulin treated group (96.0 ± 15.5 mmHg, p < 0.05). Waist to hip ratios (WHR) were similar across all groups with the insulin treated group showing the highest WHR (1.4 ± 0.4), that differed significantly from the ND and the met/RSG treated groups (ND: 1.1 ± 0.2; met/RSG: 1.1 ± 0.3, p < 0.05). As would be expected, the ND group had significantly lower fasting plasma glucose levels compared with the other 5 groups. The groups were comparable in terms of lipid profile, although only the triglyceride levels in the diet and RSG treated groups (diet: 1.6(0.9–2.3) mmol/l; RSG: 1.6(0.8–2.3) mmol/l, p < 0.05) did not differ significantly from the ND group (1.4(1.0–1.9) mmol/l, table 1).

**Table 1:** Clinical and Metabolic Characteristics of Subjects

**Variables**	**Non-Diabetic**	**Diet**	**Insulin**	**Metformin**	**Rosiglitazone**	**Met/RSG**
**Age (years)**	44.1 ± 9.9§‡*†**	48.3 ± 9.1‡***	55.6 ± 11.4	53.0 ± 10.5	52.3 ± 9.5	52.5 ± 9.0

**BMI (kg/m**^2^)	30.0 ± 5.2	29.6 ± 5.8	29.0 ± 6.2	32.0 ± 5.8	29.6 ± 5.8	31.0 ± 5.3

**Systolic (mmHg)**	117.3 ± 20.0	111.6 ± 22.9***	115.7 ± 24.9**	123.8 ± 23.5	114.1 ± 28.7**	128.4 ± 23.6

**Diastolic (mmHg)**	84.1 ± 14.6‡*	88.2 ± 14.9‡	96.0 ± 15.5***	90.3 ± 14.3**	90.9 ± 16.3	84.6 ± 13.0

**WHR**	1.1 ± 0.2§‡†	1.3 ± 0.4**	1.4 ± 0.4**	1.3 ± 0.4**	1.3 ± 0.3**	1.1 ± 0.3

**Glucose (mmol/l)**	5.5 ± 1.5§‡*†**	7.1 ± 2.8‡***	9.5 ± 3.8	9.6 ± 3.4	8.4 ± 1.9	9.4 ± 3.9

**Insulin (ng/ml)#**	20.1(10.9–23.0)‡	19.1(10.7–24.6)‡	29.9(21.2–36.6)***	20.7(10.9–27.3)	21.5(11.8–20.5)	17.1(7.6–17.2)

**LDL-C (mmol/l)**	3.2 ± 0.9§*	2.7 ± 0.8	3.0 ± 1.0	2.8 ± 0.8	2.8 ± 0.7	3.0 ± 1.4

**HDL-C (mmol/l)**	1.2 ± 0.4	1.3 ± 0.7	1.1 ± 0.4	1.1 ± 0.4	1.0 ± 0.4	1.2 ± 0.5

**TC (mmol/l)**	5.0 ± 1.0	4.7 ± 1.0	4.9 ± 1.1	4.8 ± 1.0	4.5 ± 0.9**	5.1 ± 1.2

**TG (mmol/l)#**	1.4 (1.0–1.9)***	1.6(0.9–2.3)***	1.8(1.2–2.4)	2.1(1.3–2.4)	1.6(0.8–2.3)	2.2(1.3–2.5)

**Leptin (ng/ml)#**	31.0(14.6–42.2)§*†**	18.2(5.8–23.4)‡	27.5(9.8–39.3)†**	22.5(9.6–29.6)**	16.2(5.2–19.8)	18.6(4.9–21.7)

**Adiponectin (μg/ml)#**	8.5(5.1–1.0) †**	8.6 (5.7–10.4)†**	10.4 (5.3–13.2)†**	9.1(6.0–11.3)†**	16.1(10.2–20.1)	14.3(8.9–16.1)

**CRP(μg/ml)#**	4.2(1.1–5.2)	3.8 (2.5–4.9)‡	6.3 (1.5–6.5)**	4.6(1.3–4.7)	3.7(0.8–4.6)	3.6(1.0–3.9)

**Resistin (ng/ml)#**	15.6(10.7–19.8)†	15.0(14.5–17.9)†	17.6(10.5–18.4)†**	16.1(11.3–18.1)†**	11.9(8.0–13.7)**	14.2(10.4–16.7)

**TNF-α (pg/ml)#**	5.4(3.4–7.3)***	4.7(2.9–6.2)	5.4 (2.7–7.4)*	4.2(2.8–5.3)†	5.9(4.3–6.8)**	4.5(3.3–5.6)

**Endotoxin(EU/ml)**#	4.2(3.1–5.1)§‡*†**	7.9(5.7–10.0)†	9.2 (6.6–10.7)*†**	7.5(4.6–8.7)†	5.6 (4.2–6.1)**	7.4 (4.8–9.6)

Clinical characteristics for non-diabetic (ND) and T2DM subjects on anti-diabetic therapies. Variables marked with # were log transformed prior to statistical analysis. Hence data are presented as mean ± SD unless log transformed (#) in which case they are presented as mean (interquartile range). Significance was set at p < 0.05. Therefore §denotes a significant difference in the mean values when compared with diet; ‡ denotes significance when compared with insulin; *denotes significance when compared with metformin; † denotes significance compared with RSG; ** denotes significance when compared with met/RSG.

#### Associations Between Endotoxin levels and Metabolic Factors in Subjects with T2DM

**Figure 1:**
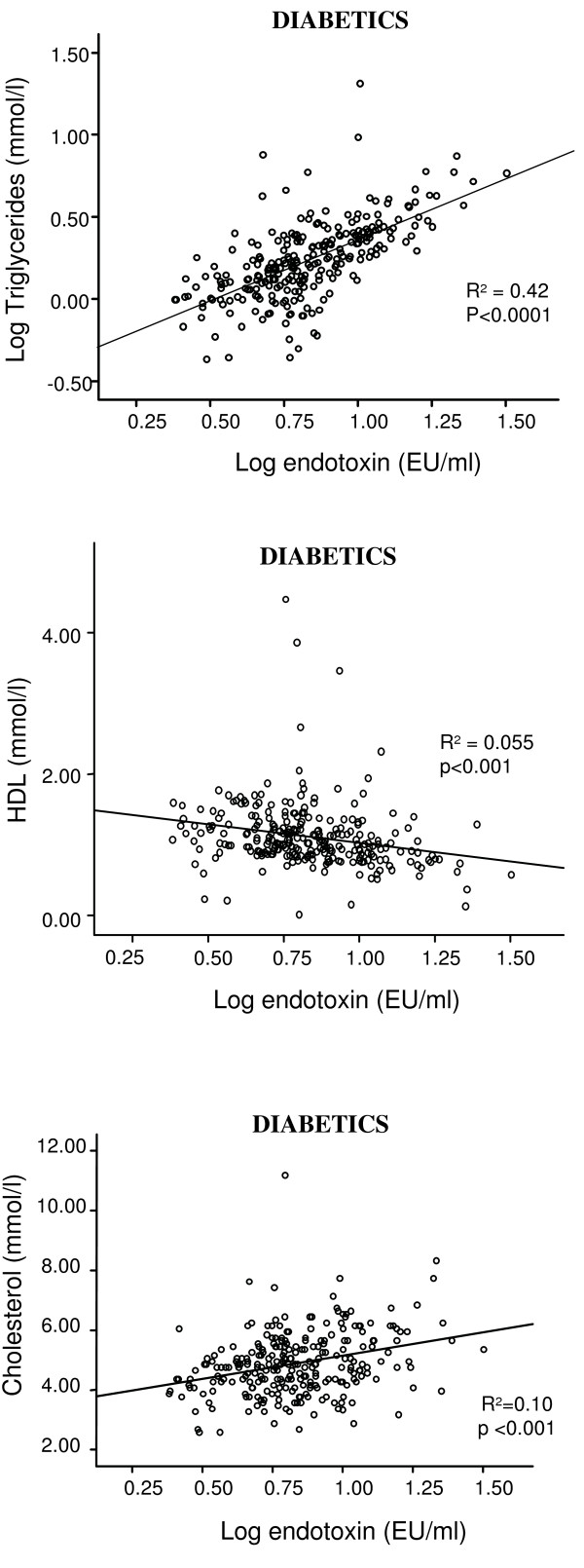
**Correlations between log fasting endotoxin (EU/ml) and a) log triglycerides (mmol/l), b) HDL (mmol/l) and c) cholesterol (mmol/l) in the whole diabetic cohort**. The lines of best fit are also shown: a) R^2 ^= 0.42, p < 0.0001, b) R^2 ^= 0.055, p < 0.001, c) R^2 ^= 0.1, p < 0.001).

**Figure 2:**
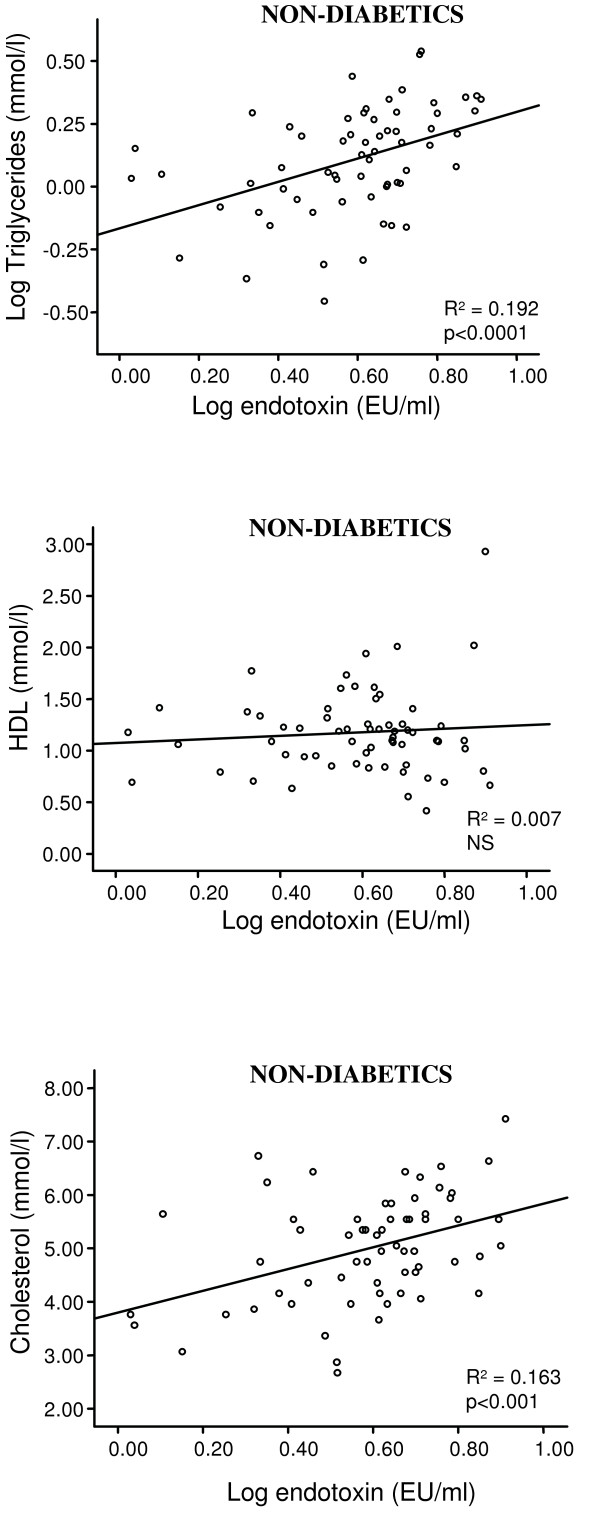
**Correlations between log fasting endotoxin (EU/ml) and a) log triglycerides (mmol/l), b) HDL (mmol/l) and c) cholesterol (mmol/l) in the non-diabetic (ND) group**. The lines of best fit are also shown: a) R^2 ^= 0.192, p < 0.0001, b) R^2 ^= 0.007, not significant (NS); c) R^2 ^= 0.163, p < 0.001).

**Figure 3:**
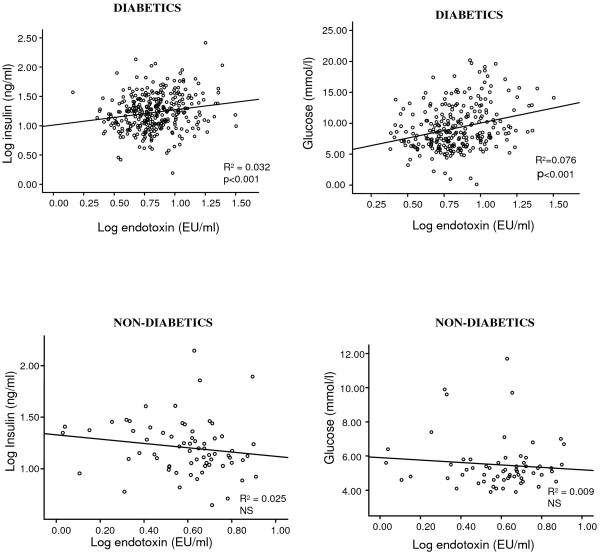
**Correlations between log endotoxin (EU/ml) and a) log fasting insulin (ng/ml) and b) glucose (mmol/l) in the whole diabetic cohort**. The lines of best fit are also shown: a) R^2 ^= 0.032, p < 0.001, b) R^2 ^= 0.076, p < 0.001. Correlations between log endotoxin (EU/ml) and c) log fasting insulin (ng/ml) and d) glucose (mmol/l) in the non-diabetic (ND) group. The lines of best fit are also shown: c) R^2 ^= 0.025, NS; d) R^2 ^= 0.009, NS.

Regression analyses revealed a significant correlation between endotoxin levels and triglycerides (R^2 ^= 0.42; p < 0.0001, figure 1a); an inverse correlation between HDL-cholesterol values (R^2 ^= 0.055; p < 0.001, figure 1b) and a significant correlation between total cholesterol levels (R^2 ^= 0.10; p < 0.001, figure 1c) in the diabetic cohort. The correlation between endotoxin levels and triglycerides, as well as cholesterol, remained even in the absence of diabetes (R^2 ^= 0.192; p < 0.0001, figure 2a; R^2 ^= 0.163; p < 0.001, figure 2c). Lastly we found a significant association between endotoxin levels and insulin (R^2 ^= 0.032; p < 0.001, figure 3a) and glucose (R^2 ^= 0.076; p < 0.001, figure 3b) in the type 2 diabetics, which was not present in the ND group (figure 3c and 3d). The rest of the analyses were not significant.

#### Circulating Endotoxin Levels in ND and Treated T2DM Subjects

**Figure 4:**
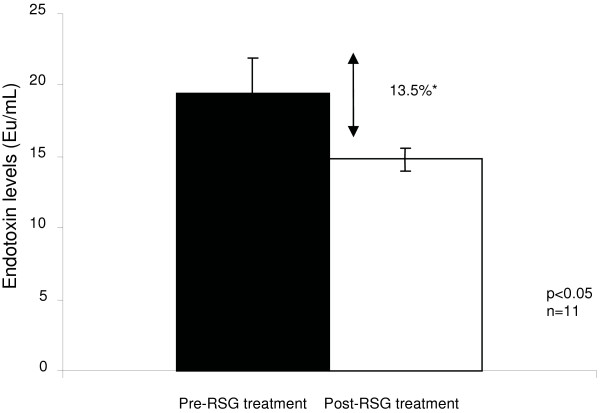
The mean levels of endotoxin (EU/ml) in sera from T2DM patients, pre and post RSG treatment (n = 11, p < 0.05*).

Serum endotoxin levels were lowest in the ND group (4.2(3.1–5.1) EU/ml, table 1), with the insulin treated cohort exhibiting the highest levels (9.2(6.6–10.7) EU/ml, table 1). Only the RSG treated group showed significantly lower endotoxin levels than all of the other anti-diabetic therapies (RSG: 5.65(4.2–6.1) EU/ml, table 1). Compared with baseline values, endotoxin levels showed a 13.5% decrease post treatment with RSG (Figure 4, p < 0.05).

#### Circulating Adipocytokine Levels

With regard to adipocytokines, the leptin levels of the ND group (31.0(14.6–42.2) ng/ml) were significantly higher compared with the treated type 2 diabetic subgroups (table 1). Adiponectin levels in the RSG (16.1(10.2–20.1) μg/ml) and met/RSG (14.3(8.9–16.1) μg/ml) treated groups were significantly higher than all the other groups, with the RSG group showing almost a two fold increase in adiponectin levels compared with the ND (8.5(5.1–10.0) μg/ml, table 1). The mean resistin levels of the groups were comparable, except for the RSG group which showed the lowest resistin levels overall (11.9(8.0–13.7)ng/ml, p < 0.05, table 1). CRP levels were highest in the insulin treated diabetics (6.3(1.5–6.5) μg/ml, p < 0.05, table 1), whilst TNF-α levels were significantly higher in the RSG treated group (5.9(4.3–6.8) pg/ml, p < 0.05, table 1).

## Discussion

Recent studies have implicated a role for adipose tissue as a site of systemic inflammation, thus providing a direct link between obesity and the associated state of chronic sub-clinical inflammation [2]. One principal source for inflammatory risk may occur via the gastrointestinal tract (GIT), as previous studies have determined that endotoxin can activate the innate immune response within adipose tissue [16,21,22,26,27]. Such studies signify a potential role for gut flora related induction of innate immunity and circulating endotoxin in the pathogenesis of obesity induced T2DM.

The findings from this study have highlighted that subjects with T2DM had significantly elevated levels of endotoxin compared with BMI matched ND subjects. Hence, these studies have affirmed previous findings in Caucasian populations whilst increasing the studied subjects' numbers four fold [21]. Our findings also demonstrated that insulin treated subjects exhibited the highest circulating endotoxin levels of all the cohorts examined; additionally, both insulin and glucose were shown to correlate significantly with endotoxin levels in the diabetic cohort but not in the control group. As such, our current and previous data have highlighted an association between insulin and endotoxin, suggesting a mechanism that hyperinsulinemia/insulin resistance may lead to increased absorption of endotoxin through the GIT [22,28]. Whilst the findings of the present study support the endotoxin/gut absorption hypothesis, it has not determined a causal effect and further studies would be required to examine this theory. Therefore, endotoxin may act as an inflammatory mediator in T2DM [21,22]. However our understanding of the relationship between glucose and endotoxin is less clear, as previous studies show no correlation between glucose levels and endotoxin [21], with Pederson and colleagues observing only a moderate increase in glucose levels when subjects underwent endotoxin infusion [29]. Conversely, severe human sepsis is known to instigate a hypermetabolic stress response, which includes hyperglycemia and impaired glucose tolerance [30]. Studies in T2DM patients have demonstrated that elevated glucose levels interfere with macrophage function, suppressing the bactericidal capacity of leukocytes [30], whilst murine studies have shown elevated glucose levels cause adverse effects on jejunum motility [31]. As such, hyperglycemia may make patients more susceptible to further infection – as observed in T2DM – as well as potentiating endotoxin absorption through inhibition of gut motility. Although patients with septic shock have considerably higher endotoxin levels (10 – 50 fold) than type 2 diabetics [32], subjects with cirrhosis are comparable with obese and T2DM subjects [33]. Therefore an association between glucose and endotoxin would suggest a role for endotoxin as an inflammatory mediator [34]. Our current findings have identified a clear, positive correlation between endotoxin and glucose serum levels [21]. The apparent discrepancies between our study and previous findings may have arisen due to the large size of the cohort we examined. Therefore the relationship between endotoxin and glucose should be investigated through subsequent studies to corroborate our findings.

The strong positive correlation identified between endotoxin and triglyceride levels is also of interest, noted in both the T2DM subjects and ND group. It is well established that subjects with obesity and T2DM have high serum triglyceride and low HDL [35,36]. Studies have indicated that HDL has a protective role against inflammation and the effects of endotoxin [37,38]. Low levels of HDL are associated with low levels of sCD14 [39], which corresponds with data showing that LPS binds to HDL in the presence of sCD14 and LPS binding protein (LBP) [40-42], and enzymes involved in the presentation of LPS to sCD14. This would imply that HDL is potentially involved in the immunological response to LPS. In contrast, serum triglyceride levels are directly stimulated by LPS [43] and are correlated with sCD14 [44]. Furthermore, it may act as a carrier of bacterial antigens and also facilitate their clearance [45,46]. These findings would have obvious implications in obese, T2DM patients.

Upon comparison of the different treatment regimes, only the RSG group showed circulating endotoxin levels significantly lower than all of the other anti-diabetic therapies. The insulin treated cohort showed the highest circulating levels of endotoxin, which advocates the theory that insulin may promote increased endotoxin absorption from the GIT [22]. Furthermore, all of the cohorts on anti-diabetic drug treatments, metformin, RSG and met/RSG, showed significantly lower levels of endotoxin than insulin – with the RSG treated group exhibiting the most dramatic decrease. Upon further analysis of pre and post RSG treated T2DM subjects, there was a 13.5% decrease in endotoxin levels over a 6 month period. These results confirm previous studies to show that RSG significantly reduces endotoxin in T2DM patients [21], whilst also indicating that RSG has a more significant effect on endotoxin levels than other anti-diabetic therapies. Therefore, these findings may explain some of the anti-inflammatory properties of the TZDs.

In addition, RSG and met/RSG treated subjects both resulted in elevated adiponectin levels that, whilst comparable to one another, were significantly higher than all the other treated groups, including the ND group. These data affirm findings by Rosenstock and colleagues and support their suggestion that the observed increase in adiponectin is due to RSG, as the TZDs have been shown to consistently increase circulating adiponectin [47].

Assessment of other adipocytokines showed that leptin levels were highest amongst the ND group and did not correlate with endotoxin in all groups. However, to date, the findings regarding leptin are conflicting. Patients with sepsis are known to have elevated levels of leptin [48], yet several studies have failed to demonstrate an increase in leptin with endotoxin infusion [49,50], whilst studies in mice and hamsters show increased leptin at the serum and mRNA level with endotoxin administration [51,52]. Such differences may have arisen from methodological reasons, as our study measured the relationship between leptin and endogenous endotoxin serum levels, as opposed to the effects on leptin as a result of exogenous endotoxin administration. Furthermore, within this study, the diabetic drugs may have decreased leptin concentration, altering the determined findings [15,53].

In summary, both ND and diabetic subjects showed significant associations with endotoxin and triglycerides and total cholesterol. T2DM subjects demonstrated an inverse correlation between endotoxin and HDL-cholesterol and positive correlations between endotoxin and glucose and endotoxin and insulin. As such, endotoxin may mediate various metabolic changes in people with T2DM in response to an increasing insulin resistant state, which is also consistent with chronic systemic low-grade inflammation. Our data suggest that the sub-clinical inflammation observed in T2DM patients may, in part, be derived from commensal bacteria [54]. Whilst clinical treatment of T2DM implies that RSG has the most favorable effect on overall endotoxin reduction and adipocytokine profiles in the T2DM patients, the present studies have simply identified associations and clearly additional randomized studies are required to determine the cause and effect via further examination of the role of endotoxin and the insulin resistant state. However current data suggest that endotoxin may represent an important mediator for inflammatory related metabolic risk.

## Competing interests

The authors declare that they have no competing interests.

## Authors' contributions

OA for the design, statistical analysis and drafting of the manuscript; NA for the drafting and revising of the manuscript; KA for the acquisition and interpretation of data; NdS for performing the endotoxin assays; SS for the statistical analysis and interpretation of data; PM and SK for the concept, interpretation of data and intellectual input; AH for the design and concept, manuscript development and final revision of the paper.
